# Computational biomechanical modelling of the rabbit cranium during mastication

**DOI:** 10.1038/s41598-021-92558-5

**Published:** 2021-06-23

**Authors:** Peter J. Watson, Alana C. Sharp, Tarun Choudhary, Michael J. Fagan, Hugo Dutel, Susan E. Evans, Flora Gröning

**Affiliations:** 1 Department of Engineering, University of Hull, Hull, HU6 7RX UK; 2 Institute of Life Course and Medical Sciences, University of Liverpool, Liverpool, L7 8TX UK; 3 School of Earth Sciences, University of Bristol, Bristol, BS8 1TQ UK; 4 Centre for Integrative Anatomy, Department of Cell and Developmental Biology, University College London, London, WC1E 6BT UK; 5 Aberdeen Centre for Arthritis and Musculoskeletal Health, School of Medicine, Medical Sciences and Nutrition, University of Aberdeen, Aberdeen, AB25 2ZD UK

**Keywords:** Computational models, Musculoskeletal system, Biomedical engineering

## Abstract

Although a functional relationship between bone structure and mastication has been shown in some regions of the rabbit skull, the biomechanics of the whole cranium during mastication have yet to be fully explored. In terms of cranial biomechanics, the rabbit is a particularly interesting species due to its uniquely fenestrated rostrum, the mechanical function of which is debated. In addition, the rabbit processes food through incisor and molar biting within a single bite cycle, and the potential influence of these bite modes on skull biomechanics remains unknown. This study combined the in silico methods of multi-body dynamics and finite element analysis to compute musculoskeletal forces associated with a range of incisor and molar biting, and to predict the associated strains. The results show that the majority of the cranium, including the fenestrated rostrum, transmits masticatory strains. The peak strains generated over all bites were found to be attributed to both incisor and molar biting. This could be a consequence of a skull shape adapted to promote an even strain distribution for a combination of infrequent incisor bites and cyclic molar bites. However, some regions, such as the supraorbital process, experienced low peak strain for all masticatory loads considered, suggesting such regions are not designed to resist masticatory forces.

## Introduction

The remodelling of bone has been proposed to follow the “mechanostat” model of bone regulation^1,2^, whereby bone is either formed or resorbed (i.e. remodelled) in response to mechanical strains. Furthermore, it has been suggested that bone structure is optimised in response to the loading environment associated with normal activity^3^. This hypothesis has been supported through in silico modelling of weight-bearing limbs, which found that bone density correlated with the strain distribution generated during common daily locomotive activities^4,5^. However, this functional relationship is more complex when considering the skull. Although it may be presumed that bone mass is regulated in response to masticatory loads, apparently robust sections of the cranium often experience low strain during biting^6–8^. This finding has led to the suggestion that while some regions are optimised to resist masticatory loads, others evolved in response to infrequent non-masticatory traumatic loads^6^ or demands not related to feeding. Therefore, the loading environments driving structural optimisation in the cranium remain a subject of debate.

**Figure 1:**
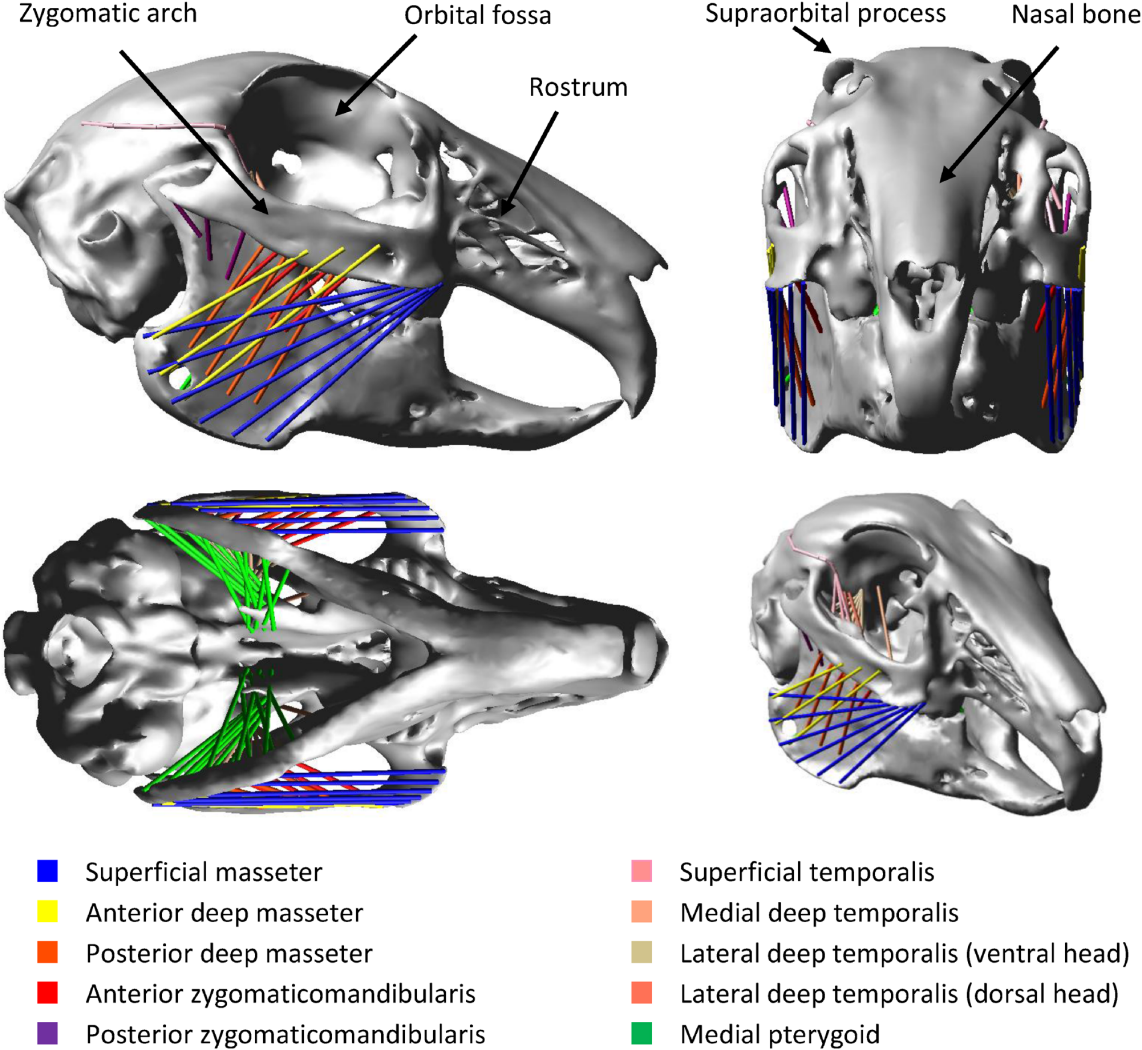
The multi-body dynamics model of the rabbit skull showing the strands used to represent the masseter, zygomaticomandibularis, temporalis and pterygoid muscles. The lateral pterygoid was also included in the model, but is not visible in the depicted views.

The functional relationship between the loading regime and cranial structure is of particular interest in species that have unique structural features, or when form-function information is of clinical relevance. Mammals of the family Leporidae (e.g. rabbit) fulfil both criteria. Firstly, the adults have a unique fenestrated rostrum (Fig. 1), which has led to numerous hypotheses as to its biomechanical function. For example, the latticing of the maxilla is hypothesised to increase the efficiency of speedy locomotion by reducing weight^9^. However, Moss and Feliciano^10^ argued that these fenestrations are rather related to the lack of transmission of masticatory forces through the lateral aspect of the rostrum. They proposed that the more vertical orientation of the ramus of the mandible in some leporids redirects incisal forces along the dorsal and ventral aspects of the rostrum, and away from the lateral side. They therefore postulated that the increased fenestration of the rostrum was a response to “phylogenetic unloading” and not linked to posture or locomotion. However, faster, saltatorial species such as hares and jackrabbits belonging to the genus *Lepus*, have more extensive fenestration^11^ and a more pronounced facial tilt^12,13^ compared to generalists including cottontail and pygmy rabbits (e.g. *Brachylagus* and *Sylvilagus*), suggesting that there may be a correlation between posture, locomotion and rostral fenestrations as well as mastication. Secondly, understanding the healthy rabbit masticatory system is a prerequisite for diagnosis and treatment of dental disease in rabbits^14^, which is one of the most common diseases reported in the rabbit, but the exact cause remains unknown despite several hypotheses^14–16^. In addition, this understanding can facilitate the development of in silico models simulating the healthy rabbit masticatory system, which subsequently has the potential to help replacement, refinement, and reduction (3Rs) of experiments using rabbits in biomedical and veterinary research.

A direct functional relationship between masticatory forces and bone structure has been observed experimentally in the cranium. For example, experiments have shown that a hard food diet causes an increase in bone thickness and biomineralisation of the hard palate^17,18^ and in biomineralisation of the zygomatic arch^19^, when compared to soft food diets. This presumably corresponds to the higher masticatory muscle activity and associated bone strains when rabbits chew harder foods^20,21^. However, the complexity of this functional relationship is highlighted by the observation that some regions of the cranium, such as the neurocranium, display minimal structural change in response to an altered diet^18,19^.

Studies measuring strain in the cranium due to mastication have observed that peak bone strains decrease when moving from the maxilla towards the neurocranium and circumorbital region^22,23^. A similar strain gradient is likely to characterise the rabbit cranium^19^, however this is yet to be investigated as current data only provide strains in localised regions. Also, as the rabbit consumes large food items through gnawing by the incisors to break off small pieces, and then reduction of these pieces through molar biting^24^, it is also unknown how these different bite modes influence the peak masticatory strains across the whole cranium. This is difficult to test experimentally with strain gauge measurements for the following reasons: firstly, implanting strain gauges onto bones is an invasive procedure and can affect biting behaviour^25^; secondly, it is possible to apply only a limited number of gauges to a cranium, so that strain can be measured only at a few points.

Finite element analysis (FEA) is an in silico technique that provides an alternative to strain gauge measurements when investigating how the cranium deforms under certain loading conditions. FEA has been used to analyse cranial stresses and strains resulting from masticatory loads in a wide range of taxa^26–35^. In addition, FEA can be integrated with multi-body dynamic analysis (MDA) to inform the FEA with kinetic data associated with various forms of mastication^29,34,36,37^. MDA – FEA integration also allows the prediction of strains associated with a range of bites, across the entire skull. Applying such in silico techniques to the rabbit cranium would therefore provide the opportunity to characterise the distribution of peak strains associated with different modes of biting, and directly assess their impact on the loading in the rostrum.

In this study we use in silico modelling to investigate the distribution of peak bone strains associated with differing modes of biting (incisor and molar biting) and bite location, in the rabbit cranium. Through a combination of MDA and FEA methods, the study has the following aims: 1. to analyse the distribution of peak strains across the cranium resulting from a wide range of bites; 2. to investigate which mode of biting (i.e. incisor or molar biting) has the greatest influence on the peak strains; 3. to assess the extent to which masticatory forces are transmitted through the fenestrated rostrum.

## Results

Musculoskeletal loading associated with processing hay through incisor and molar biting was computed using an MDA model of a European wild rabbit (*Oryctolagus cuniculus*) skull^38^ (Fig. 1). The MDA model was used to create loading regimes for unilateral biting on both incisors, and unilateral biting on every tooth (left and right) along the molar row. This generated a total of 12 separate bite loading regimes (for further details of the MDA modelling see Methods).

A detailed FE model of the rabbit cranium was created with a very high element density to accurately represent the intricate features of both hard (bone and teeth) and soft (sutures, periodontal ligament [PDL] and pulp) materials. A total of 12 separate FE analyses were conducted, one for each of the bite loading regimes. The strains in the cranial bone are displayed in terms of the largest strains generated throughout the structure by all bites, which was achieved by selecting the largest strain in each element from any of the separate bites. These are referred to as peak strains. For detailed information on how the peak strains were calculated, see Methods.

**Figure 2:**
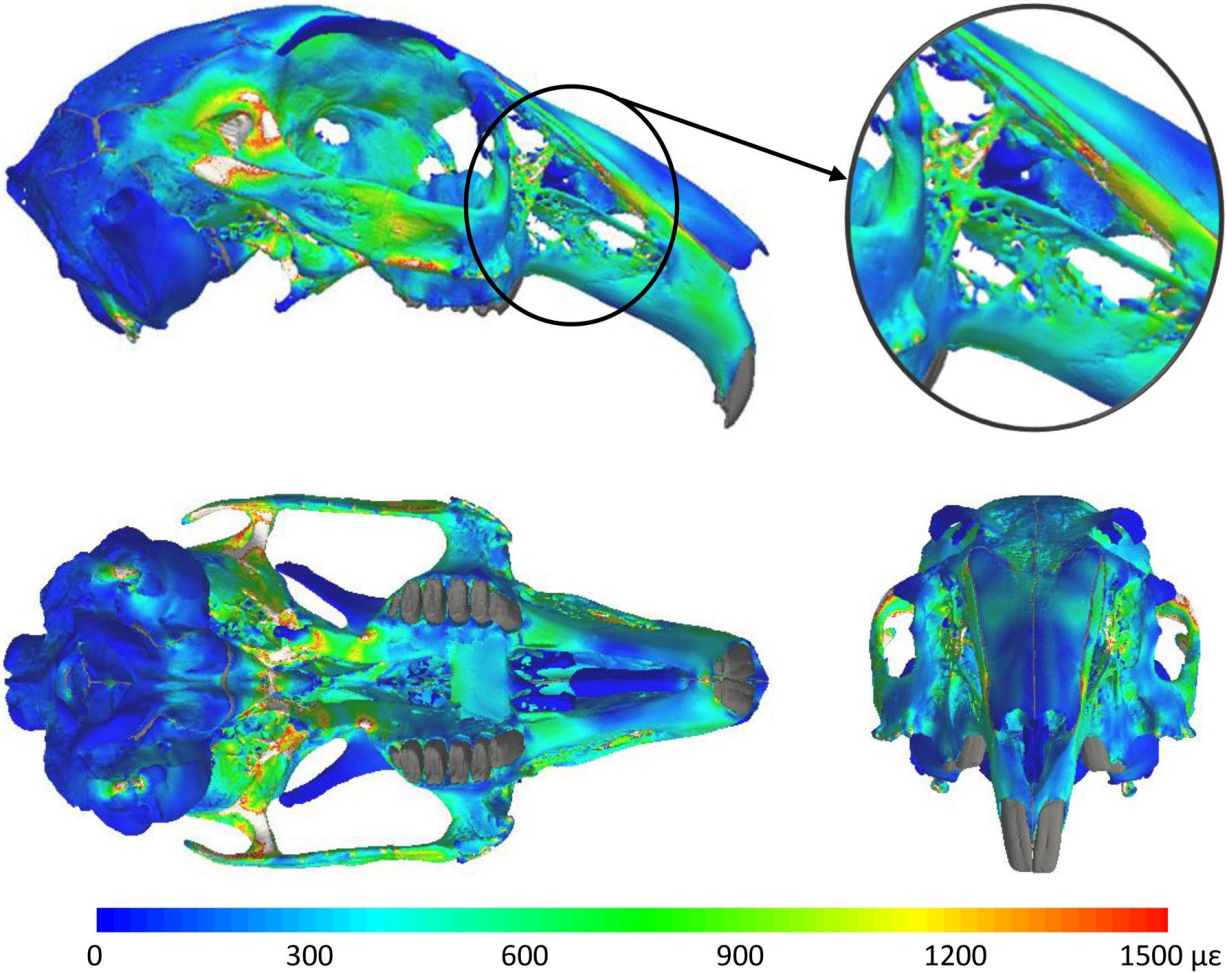
Peak von Mises strain generated throughout the cranium by 12 separate unilateral bites (2 incisor and 10 molar bites). The plots show the largest strain in each element from any of the separate bites. The insert shows the strains in the rostrum. Regions in light grey represent strains above 1500µɛ, teeth and sutures are shown in dark grey.

**Figure 3:**
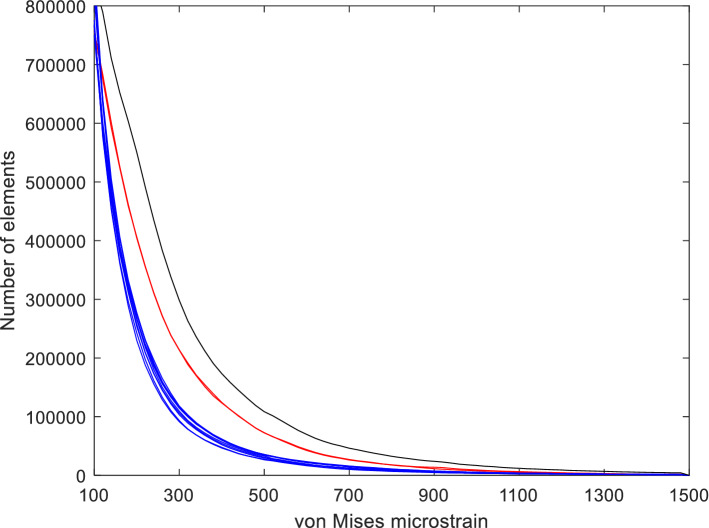
The number of elements that experienced a specific strain magnitude using a frequency bin size of 20 µε. The plot shows the strains from all 12 separate unilateral bites (2 incisor bites [red lines] and 10 molar bites [blue lines]) and the cumulative peak strains for all bites (black line).

A plot of the peak von Mises strain (Fig. 2) in each element produced a distribution with two main concentrations of large values: one around the temporomandibular joint (TMJ) that extends to the glenoid fossa and posterior region of the zygomatic arch; and another in the basioccipital bone that travels to the alisphenoid bone, towards the glenoid fossa. High peak strains were also located in sporadic regions of the cranium, such as the rostrum and zygomatic arch. In contrast, the distribution of low peak strains was primarily located in the nasal bone, supraorbital process, frontal bone, parietal bone and posterior regions of the cranium. The combination of the peak bone strains from all bites lead to more elements experiencing higher strains than incisor and molar biting in isolation, thus generating a more uniform strain distribution across the cranium (Fig. 3).

**Figure 4:**
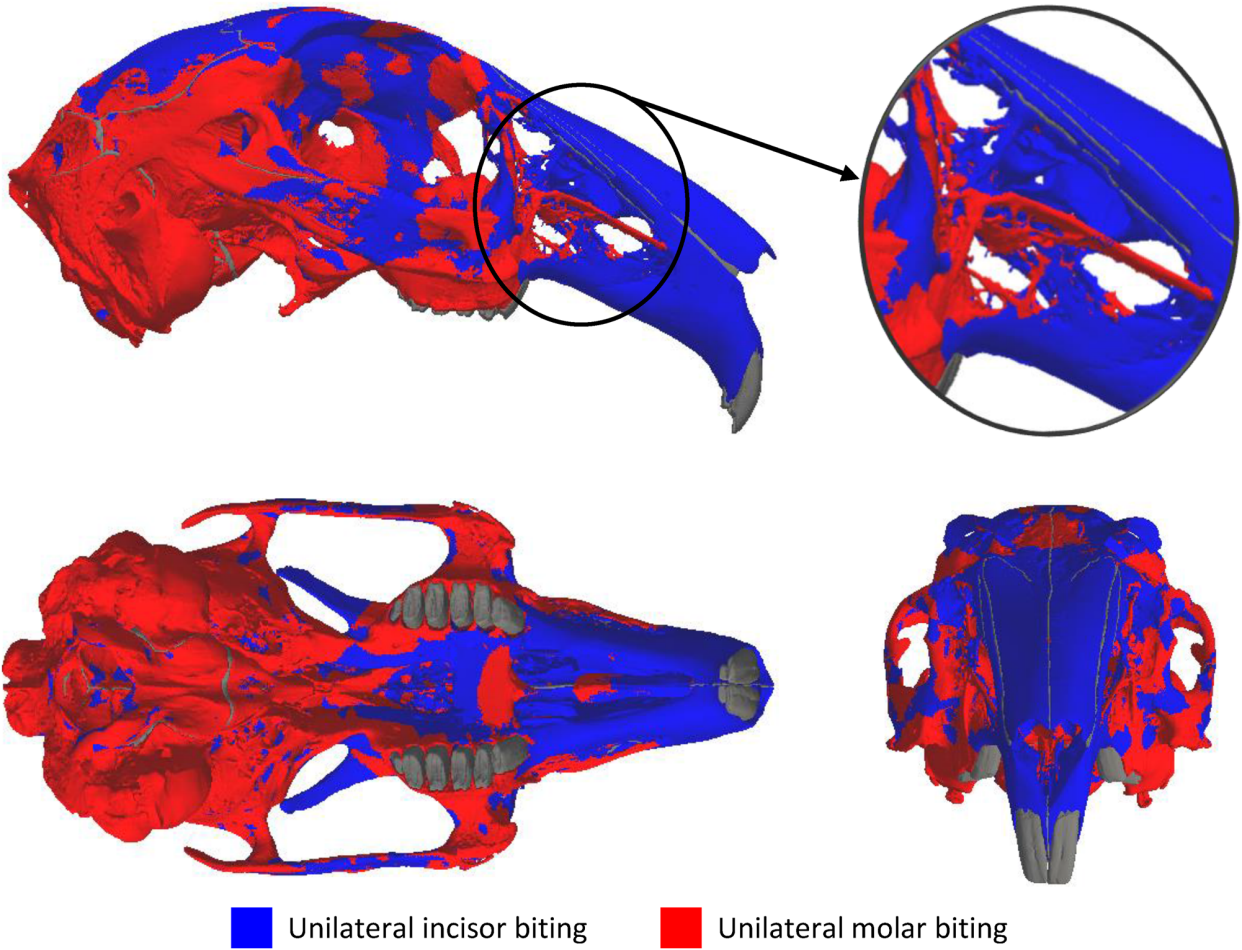
Dominant modes of biting over 12 separate unilateral bites (2 incisor and 10 molar bites). The plots show which mode of biting produced the largest strain in each element: blue represents regions where unilateral incisor biting produced the largest strains; red represents regions where unilateral molar biting produced the largest strains. The insert shows the rostrum. The teeth and sutures are shown in dark grey.

Molar biting was the greatest contributor to the peak strains shown in Fig. 2, accounting for the highest values in 60.0% of the bone volume. The peak strains in the premaxilla, frontal bone and parietal bone were predominantly produced by incisor biting, while peak strains in the alveolar process, squamosal and tympanic bulla were caused by molar biting (Fig. 4). Peak strains in other regions, such the zygomatic arch, hard plate and orbital fossa (anterior to the foramen for the optic nerve), were generally caused by a combination of incisor and molar biting.

**Figure 5:**
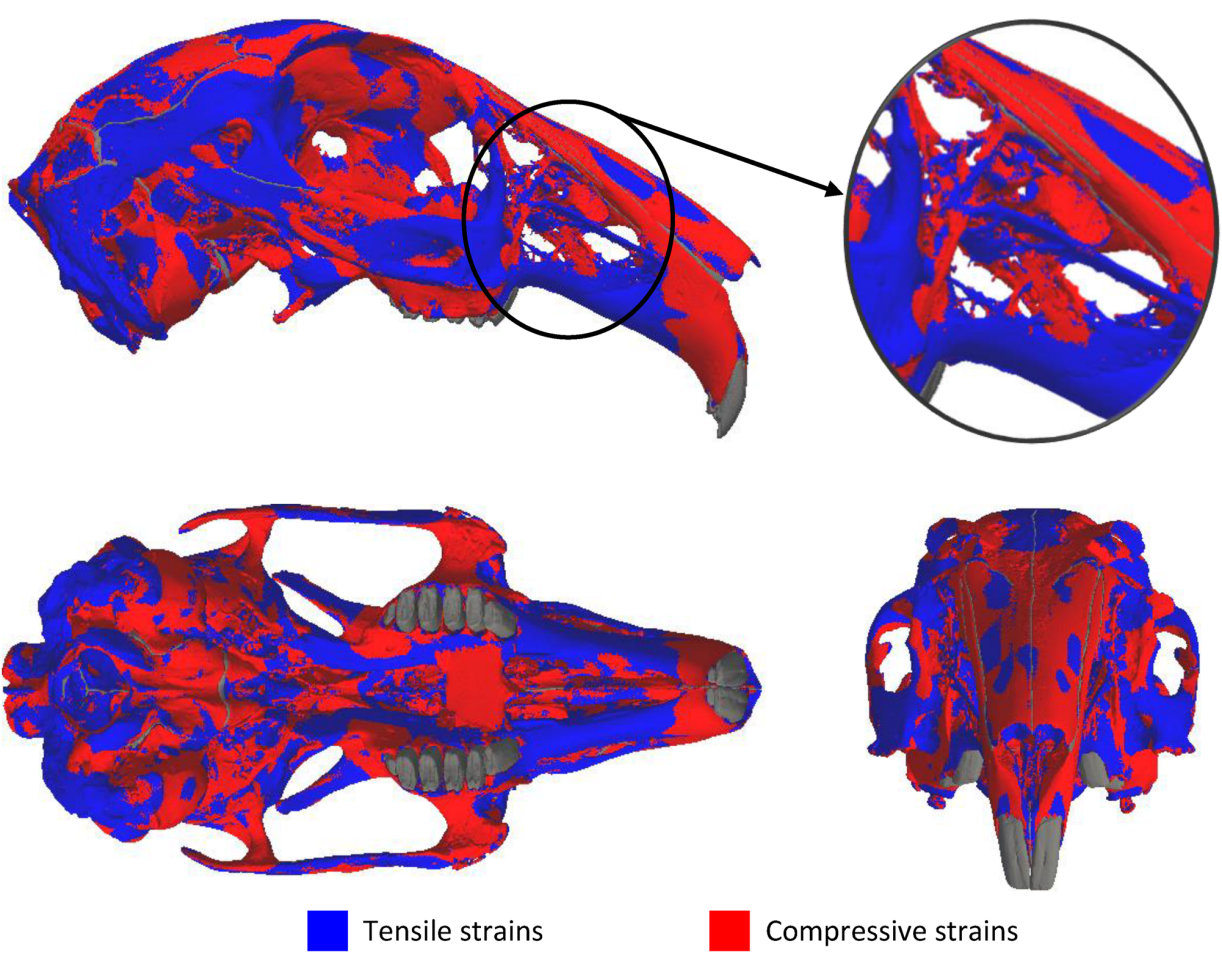
Dominant principal strains over 12 separate unilateral bites (2 incisor and 10 molar bites). The plots show which principal strain produced the largest strain in each element: blue represents the regions of the cranium where tensile strains are greater than compressive strains (i.e. tensile strains are dominant); red represents regions where compressive strains are greater than tensile strains (i.e. compressive strains are dominant). The insert shows the rostrum. The teeth and sutures are shown in dark grey.

As the cranium deforms under loading, it experiences tensile, shear and compressive strains. The peak principal strain in each element across the cranium was observed to be a relatively equal distribution of compressive and tensile strains (Fig. 5). Compressive strains occurred in 53.3% of the bone volume, while tensile strains accounted for 46.7% of the bone volume, with some regions predominately experiencing a single dominant principal strain (such as compressive strains in the nasal bone) and other regions (such as the zygomatic arch) experienced a combination of principal strains.

Further analyses to test the sensitivity of the FE model found that lowering the stiffness of the PDL and sutures caused changes to the local peak bone strain values, however these differences were limited in magnitude and did not extend to the whole structure (Supplementary Information Figs. S2 and S4).

## Discussion

Through the combination of MDA and FEA methods this study has, for the first time, predicted the distribution of peak bone strains associated with a wide range of bites in the rabbit cranium. The MDA simulations considered the processing of hay since this is one of the toughest foods in the rabbit diet, and is known to require more chewing investment and duration^39^. The peak strains generated by all incisor and molar bites show a varied strain distribution across the cranium, with the bones of the masticatory system (maxilla and temporal bone) generally experiencing larger strain magnitudes when compared to other regions of the cranium, such as the supraorbital process, frontal bone and posterior regions of the cranium (Fig. 2) (Aim 1). The location of low strain magnitudes agrees with experiment observations of cranial strains during mastication^22,23^.

The temporal fascia has been reported to relieve strain in the zygomatic arch^40^. Therefore, it is possible that zygomatic strains could be lower in vivo*,* but this remains speculative as the authors are not aware of any experimental studies of temporal fascia function or descriptions of temporal fascia insertion in the rabbit. In addition, the posterior region of the cranium may have experienced additional strains if the MDA model had considered the actions of the neck muscles. However, as the neck muscles act posterior to the constraints at the TMJ it is unlikely that their inclusion would affect the strains in the anterior regions^35^.

Analysis of the peak strains generated by the two modes of biting showed that no single mode was dominant (Aim 2) (Fig. 4). Although incisor biting was found to be the largest contributor to the peak strains in the premaxilla, regions posterior to the premaxilla experienced peak strains from a combination of the two bite modes. The latter is most noticeable in the zygomatic arch, a region known to respond to masticatory forces^19^, where the activation of the masseter muscles during both incisor biting and molar biting cause peak strains. As no single mode of biting was dominant, the combination of peak bone strains from all bites lead to a more uniform strain distribution across the cranium (Fig. 3). In addition, the distribution of both peak compressive and tensile strains (Fig. 5) suggests that the cranium may be adapted to resist a combination of compressive and tensile loads. These findings are in line with previous modelling studies in the reptile *Sphenodon*^29^ and demonstrate that, when investigating skull biomechanics, analyses should consider all bite locations and relevant modes of mastication.

The peak strain distribution produced by both modes of biting showed that the fenestrated rostrum does transmit forces from masticatory loads (Aim 3). The peak strains experienced in the struts on the lateral aspect of the rostrum were generally similar in magnitude to other regions experiencing high peak strain (Fig. 2). In addition, Fig. 4 demonstrates that peak strains in most of the posterior struts were predominately generated by molar biting, while the dorsal and ventral regions of the rostrum are strained more by incisor biting. Therefore, it is possible that the fenestrations are an adaptation to minimise mass in the rostrum while maintaining structural integrity during biting, supporting the hypothesis of Moss and Feliciano^10^. More advanced in silico modelling could utilise adaptive remodelling within the FEA to analyse the extent to which the rostrum is optimised to reflect this.

Separate layers of cranial bone in the rabbit are known to adapt differently in response to masticatory loads, for example there is regional variation in whether bone alters in thickness or biomineralisation (or even both)^17–19^. The strain magnitudes required to cause these bone adaptations is unknown, but this study has provided some insight into the magnitudes of these peak strain associated with a wide range of bites. Although there is no accompanying experimental strain data to validate the predicted peak strains in Fig. 2, the magnitudes are similar to those measured in the rabbit mandible during chewing^20^. In addition, the majority of the predicted strains are less than the estimated yield strain for the cortical bone modelled here^41^, apart from local high strain concentrations around the constraints at the TMJ, which are likely to be artefacts of constraint configuration.

In terms of the strain magnitudes, 62.5% of the cranial volume experienced peak strains above 100 µɛ, and below 1500 µɛ, which is the range suggested for normal bone remodelling, i.e. maintenance of homeostasis^1,2,42^. Consequently, when analysing static load cases, as is common in skull FE modelling, this study suggests that large regions of the skull experience strains that are capable of maintaining homeostasis. This includes the struts within the rostrum (Fig. 2), providing evidence that the fenestrations are likely adapted to dissipate masticatory forces. It should be noted that this analysis does not consider strain rate, which is known to be an additional factor in bone remodelling^43^ and can alter the strain magnitude that initiates bone formation^44^. However, the 100 –1500 µɛ range provides a benchmark figure for analysing static load cases. Interestingly, strains above 100 µɛ were observed in regions of the neurocranium (such as the orbital fossa and frontal bone), which is not thought to respond to masticatory loading^18,19^. It is possible that these regions may have a higher strain threshold for normal bone remodelling or that strain rate plays an important role in modulating remodelling in these regions. This demonstrates that conclusions on form-function relationships in the skull cannot rely solely on strain magnitudes from static load cases. Although strain gradients are frequently used to obtain information about the overall response of bone to masticatory forces, this approach does not take into account possible regional variation in the mechanism underlying bone adaptation^19^.

A high density FEA mesh was used to model the intricate hard and soft tissue structures of the rabbit cranium as accurately as possible. As with all FE modelling, element density is known to influence the accuracy of the strain distribution in cranial and mandibular modelling^45,46^, and it is known that strains in thin structures are particularly error prone if insufficient elements are modelled through the structure^47^. This is particularly important when modelling the rabbit cranium due to the thin struts in the rostrum, PDL and cranial sutures. Therefore, this study used a particularly high-density mesh (~ 41 million second order tetrahedral elements) (for information on the mesh see Methods). The FEA model included the PDL for each tooth as studies have shown that inclusion of the PDL can significantly influence alveolar bone strains^48–50^. Moreover, the shape of the PDL modelled here was determined from a µCT scan as accurately representing the morphology of the PDL has been shown to further influence bone strains^51^. In addition, anatomically detailed cranial sutures were included since studies have shown that sutures influence bone strains^52–56^. However, more studies have examined sutures in reptiles than mammals, limiting our understanding of mammalian cranial suture function. This is one of few studies that have modelled cranial sutures in a mammal.

Inevitably there are assumptions and limitations in the models. Unfortunately, the variation in density of the bone could not be determined sufficiently accurately from a µCT scan and due to the lack of any reported material properties for the bone in the rabbit skull, this study used a constant Young’s modulus value throughout the model based on those of a canine skull. Modelling of element specific material properties would provide a better representation of the true heterogenous nature of bone stiffness. Likewise, a range of material properties have been used previously to model the PDL and sutures, therefore sensitivity tests were performed to assess the influence of the soft tissues on bone strain magnitude and distribution. Changes in peak bone strains due to lowering the stiffness of the soft tissues were localised and limited in magnitude (Supplementary Information Figs. S3 and S4), therefore the peak strain distributions across the whole cranium (Supplementary Information Figs. S1 and S2) were observed to be similar to that of Fig. 2. Thus, the sensitivity of results to soft tissue elastic modulus did not affect the observations made regarding the strain distribution associated with mastication.

As customary in MDA modelling of skulls, this MDA model relied on literature to calculate the maximum isometric muscle forces, estimate the muscle activation profiles and assume jaw kinematics^38^. To improve the MDA model, further experiments are required to obtain physiological muscle data and skull kinematics, ideally during feeding on a range of foods from the same individual. This would enable the construction of an individual-specific MDA model and facilitate the validation of the model across a range of bite modes^57^. Coupled with measurements of material properties, this would create a fully validated MDA – FEA analysis of the same individual.

Such a fully validated in silico modelling would have significant potential in the replacement, refinement, and reduction (3Rs) of experiments using rabbits in biomedical and veterinary research. The rabbit is one of the most common animals used in such research^58,59^ and is often used to investigate the remodelling of bone in response to dental implants^60–63^ and impaired muscle function^64,65^. Furthermore, the integrated approach of MDA – FEA modelling could be expanded to include adaptive remodelling algorithms to predict the response of bone to the strains associated with masticatory dysfunction. Therefore, such in silico modelling has significant potential to reduce the dependency on such animal experiments.

In conclusion, this in-depth biomechanical analysis has shown the gradient of peak strains in the rabbit cranium during mastication. The cranial shape in rabbits appears to accommodate a uniform strain distribution for a combination of infrequent incisor bites and more frequent cyclic molar bites. These results also show that the rostrum plays a role in the transmission of masticatory forces, therefore it is hypothesised that the fenestrations may be optimised to facilitate this transmission while minimising bone mass. Bones of the masticatory system experience the largest peak strains. However, the lower peak strain magnitudes in other regions, such as the supraorbital process and posterior cranium, suggest these could be the result of an adaptation to resist other forms of loading (e.g. from the neck muscles or possibly more impactful loads due to locomotion), a non-mechanical adaptation, or the result of a developmental and/or evolutionary constraints.

## Methods

### Multi-body dynamic analysis

An MDA model of a European wild rabbit (*Oryctolagus cuniculus*) previously developed by Watson et al.^38^, and validated against in vivo bite force, was used to predict the musculoskeletal loading associated with incisor biting and molar biting (Fig. 1). The representation of the medial pterygoid and lateral deep temporalis (ventral head) was altered to remove the need to include muscle wrapping to facilitate the subsequent FEA. This had minimal influence on the line of action of these muscles. The line of action of the superficial temporalis and lateral pterygoid was represented by multiple strands to model the wrapping of these muscles around the cranium and/or other musculature. The MDA model contained a total of 130 individual muscle strands (65 strands on each side) to accurately model the masticatory muscles (Fig. 1). The TMJ was modelled through contact analysis which enabled movement of the jaw in all degrees of freedom (DOF). Full details of the model construction are provided in Watson et al.^38^.

A series of simulations was performed in Adams v.2021.0.1 (MSC Software Corp., United States; https://www.mscsoftware.com/product/adams) to mimic unilateral incisor biting on the right side and unilateral molar biting on each tooth along the right molar row (i.e. 1st premolar, 2nd premolar, 1st molar, 2nd molar and 3rd molar). Due to the symmetric nature of the cranium, the muscle and bite forces from the unilateral bites on the right side were reflected to create loading regimes for biting on the left side. This created a total of 12 separate bite loading regimes.

The muscle strands were modelled using the parameters of maximum isometric force, activation factor and passive tension. The muscles were activated through the application of the Dynamic Geometric Optimization (DGO) method (for a detailed description of the method see Curtis et al.^66^). The DGO method estimated the muscle forces (taking into account the instantaneous muscle strand orientations) to achieve a pre-defined jaw motion. The strands also carried a small passive tension in resistance to their elongation, which increased exponentially to a maximum of 0.1% of the maximum isometric muscle force. This passive tension did not affect muscle activations or bite forces. The DGO activated the working and balancing side muscles in a coordinated manner using the EMG recordings of Weijs and Dantuma^24^ to mimic jaw excursion associated with incisor and molar biting.

When simulating molar biting the DGO activated the muscles so the bite was modelled with the three distinct phases of a reduction bite cycle^67^: (i) opening phase, (ii) fast closing phase (closing of the jaw until it contacts the food bolus), and (iii) slow closing phase (where the food bolus is processed). A food reduction bite cycle was chosen as it is representative of the midpoint of a chewing cycle and the duration of each phase was also consistent with literature^67^. The jaw kinematics for molar biting was based on the description of Weijs and Dantuma^24^ during the processing of hay. Therefore, the jaw reached a maximal gape of 12° in the mid-sagittal plane during the opening phase, rotated ~ 5.5° to the working side in the frontal plane during the fast closing phase, and then rotated back to the midline at the end of the slow closing phase. The teeth had to overcome a resistance of 30 N in the mediolateral direction in order to return to the midline. This value is within the range observed for barley straw^68^. A maximal resistance of 30 N was defined in the vertical direction for the food bolus to be compressed completely.

Incisor biting was modelled with the same maximal gape but the DGO activated the muscles simultaneously so the jaw exhibited symmetrical rotation about the midline in the frontal plane during the fast and slow closing phases. For comparative purposes, incisor biting also simulated the processing of hay, therefore the food bolus had the same size and vertical resistance as molar biting, with the jaw returning back to a closed position at the end of the slow closing phase.

### Finite element analysis

The same rabbit cranium used to construct the MDA model was rescanned with a X-Tek HMX 160 µCT scanner (X-Tek Systems Ltd, United Kingdom), at a higher resolution of 28 µm in each direction. The scan data was segmented in AVIZO image visualisation software v.9.5. (Visualization Sciences Group, Inc. USA) to create a volumetric model of the cranium with separate volumes for the bone, teeth, pulp, anatomically detailed sutures and PDL.

A mesh of the volumetric model was created using the Meshing Workroom in AVIZO through defining a facet size (controls the size of the surface facets) and cell size (upper bound on the circumradii of the mesh tetrahedral) for each material. This ensured the thinner regions of the model (such as the PDL, sutures and the struts in the rostrum) contained sufficient elements through the structural thickness. Therefore, the final mesh (consisting of ~ 41 million second order tetrahedral elements) contained differing element densities for the bone (facet and cell size of 0.15 mm, creating ~ 14 million elements), PDL and sutures (facet and cell size of 0.05 mm, creating ~ 16.6 million elements for the PDL and ~ 8.3 million elements for the sutures), teeth (facet and cell size of 0.2 mm, creating ~ 1.9 million elements), and pulp (facet and cell size of 0.2 mm, creating ~ 269,000 elements).

The mesh was imported to ANSYS v.17.2 (ANSYS Inc., United States) and all materials defined with homogenous, isotropic, and linear elastic properties. Bone was assigned a Young’s modulus (E) value of 13.7 GPa and a Poisson’s ratio (ν) of 0.3, as has been modelled in canines and felids^35,69,70^. The PDL and sutures were modelled with E = 50 MPa^71^ (ν = 0.49) and E = 20 MPa (ν = 0.49), respectively. As this study was not concerned with the strains in the teeth, the dentine and enamel were modelled as a single structure and assigned the material properties of dentine (E = 19.89 GPa, ν = 0.31)^72^, while the pulp was modelled with E = 2 MPa and ν = 0.49^73^.

A total of 12 separate FE analyses were conducted, one for each of the bite loading regimes. A loading regime was created for each bite simulated by the MDA model, using the force calculated in each muscle strand at the point when the bite force reached a maximum value (muscle forces are presented in Supplementary Information Table S1). The muscle forces were then applied to the FE model as a nodal load at the origin of each strand. The application of point loads in the FE model did not cause local strain concentrations at any of the strand origins (as evident in Fig. 2). To replicate wrapping of the superficial temporalis, a series of paths, each consisting of truss-type elements (ANSYS LINK180), were positioned over the bone surface^35^. The link elements were positioned at the same location as the individual muscle strands used to wrap the superficial temporalis in the MDA model. The common node connecting two link elements was then attached to the cranium with a further short link element positioned perpendicular to the surface. This created links wrapping around the cranium in a comparable path and position to the MDA model. A similar method was used to represent the lateral pterygoid muscle. The link elements were defined with the same material properties as the bone to ensure they facilitated force transfer with minimal deformation.

All FE analyses constrained a node at the tip of the tooth to simulate biting in the dorsoventral direction. Molar biting constrained a node on the working side TMJ dorsoventrally and anteroposteriorly, while a node on the balancing side TMJ was constrained in all DOF. The unilateral incisor biting analyses constrained a node on the working side TMJ in all DOF, and a node on the balancing side TMJ in the dorsoventral and anteroposterior directions. The exact location of these nodes was taken as the point of contact between the teeth and food bolus, and the mandible and TMJs, as determined by the MDA simulations. These constraint configurations were based on the stability of the working and balancing side TMJs as determined by the MDA simulations. This enabled the cranium to deform laterally and reduced the risk of artefacts from over-constraining the FE analyses.

Due to the high-density mesh, all FE analyses were solved using High-Performance Computing facilities at the University of Hull. The mesh was converted to a vtu-file using a custom-built script in Python v3.7.3^74^ so that it could be visualised in Paraview v5.0.1^75^. Post-processing of the FE analyses initially exported the strains generated by each bite in separate element tables. To determine the peak strains generated throughout the cranium by all bites, the element tables for the bites were initially imported to MatLab v2019a (The MathWorks, Inc., United States) and a custom-built script was used to create a cumulative peak element strain table by selecting the largest strain in each element from any of the separate bites. This cumulative peak element strain table was then combined with the vtu-file of the mesh in Python and finally imported to Paraview, so that the distribution of the cumulative peak element strains could be visualised across the cranium. In addition, a custom-built script in MatLab also determined which mode of biting produced the peak strain recorded in each element. This information was then combined with the vtu-file of the mesh in Python and subsequently imported to Paraview, so that the distribution of the dominant bite mode could be visualised across the cranium. A similar process was also used to determine and visualise the dominant principal strains over all bites.

The FE analyses were evaluated by considering von Mises strain because it has been employed previously to assess skull biomechanics^29,31,35,54^. In addition, von Mises strain is convenient because it is a scalar function combining the three principal strains, is related to the von Mises failure criterion, and is useful for comparing the performance of complex three-dimensional geometries.

## Supplementary Information

Supplementary Information.
