# Supplementary material for: Computational biomechanical modelling of the rabbit cranium during mastication
Source: Sci Rep. 2021 Jun 23;11:13196. doi: 10.1038/s41598-021-92558-5 (PMC8222361; doi:10.1038/s41598-021-92558-5)
Supplement: Supplementary file 1 — Supplementary Information. [file 41598_2021_92558_MOESM1_ESM.pdf]

Computational biomechanical modelling of the rabbit cranium during mastication - Supplementary Information

Peter J. Watson, Alana C. Sharp, Tarun Choudhary, Michael J. Fagan, Hugo Dutel, Susan E. Evans, and Flora Gröning

To investigate the influence of periodontal ligament (PDL) stiffness on bone strains across the cranium, all finite element analyses (one for each of the incisor and molar bites) were re-run with a PDL Young's modulus (E) of 8 MPa[1] and Poisson's ratio (ν) of 0.49.

The distribution of peak strains in the bone when modelling the PDL with E = 8 MPa (Fig. S1) was comparable with that of the peak strains predicted when modelling E = 50 MPa.

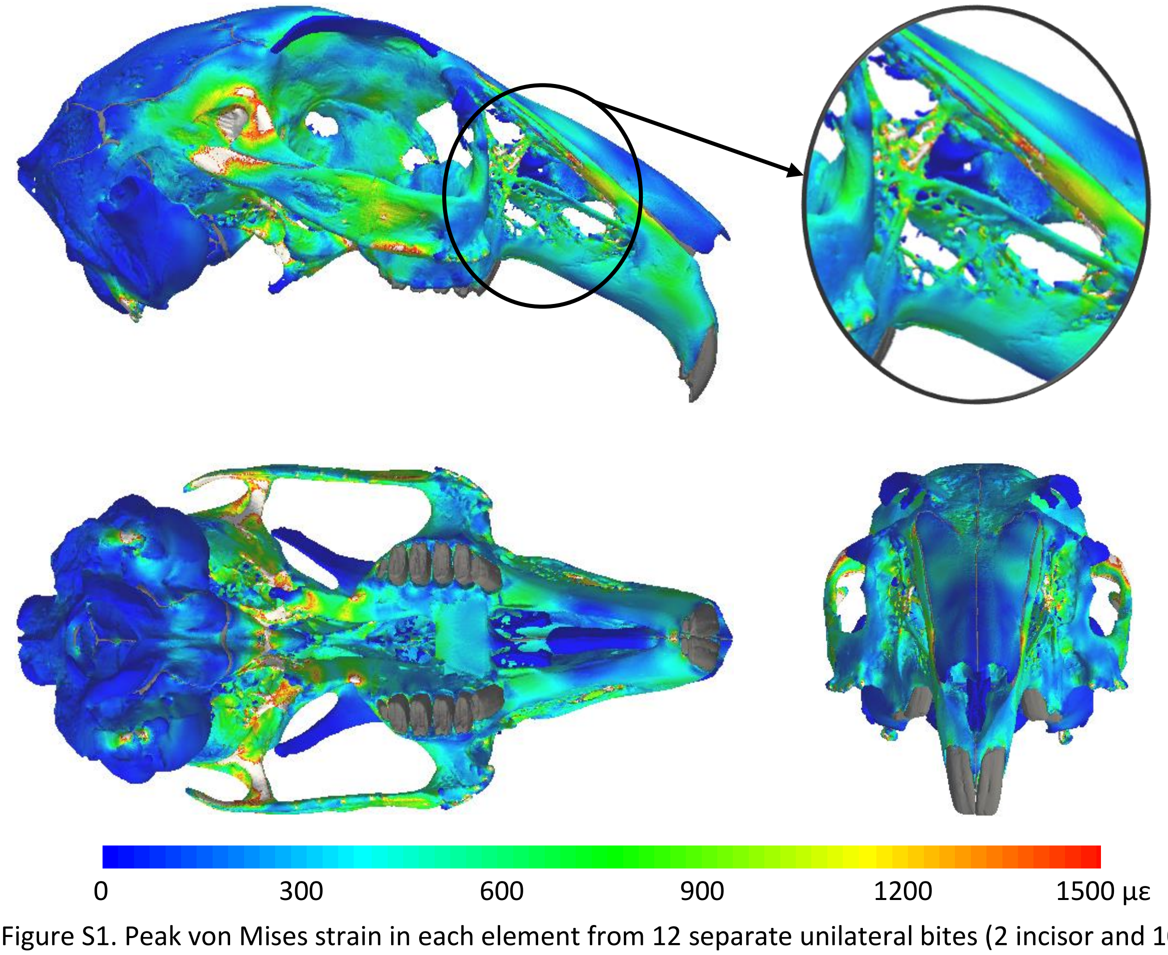

Figure S1. Peak von Mises strain in each element from 12 separate unilateral bites (2 incisor and 10 molar bites) when modelling the PDL with E = 8 MPa (ν = 0.49). The plots show the largest strain in each element from any of the separate bites. The insert shows the rostrum. Regions in light grey represent strains above 1500 με, teeth and sutures are shown in dark grey.

1. Schrock, P., Lüpke, M., Seifert, H., Borchers, L. & Staszyk, C. Finite element analysis of equine incisor teeth. Part 1: Determination of the material parameters of the periodontal ligament. *Vet. J.* **198,** 583–589 (2013).

Comparison of the difference in peak strains when altering E from 50 MPa to 8 MPa, showed that the location of these differences was limited to regions around the tooth sockets (Fig. S2). The magnitude of these differences was generally below 200 με. The sign of the difference when lowering E to 8 MPa (i.e. whether strain increased or decreased) was varied. However, the remaining regions of the cranium experienced little change in peak strains.

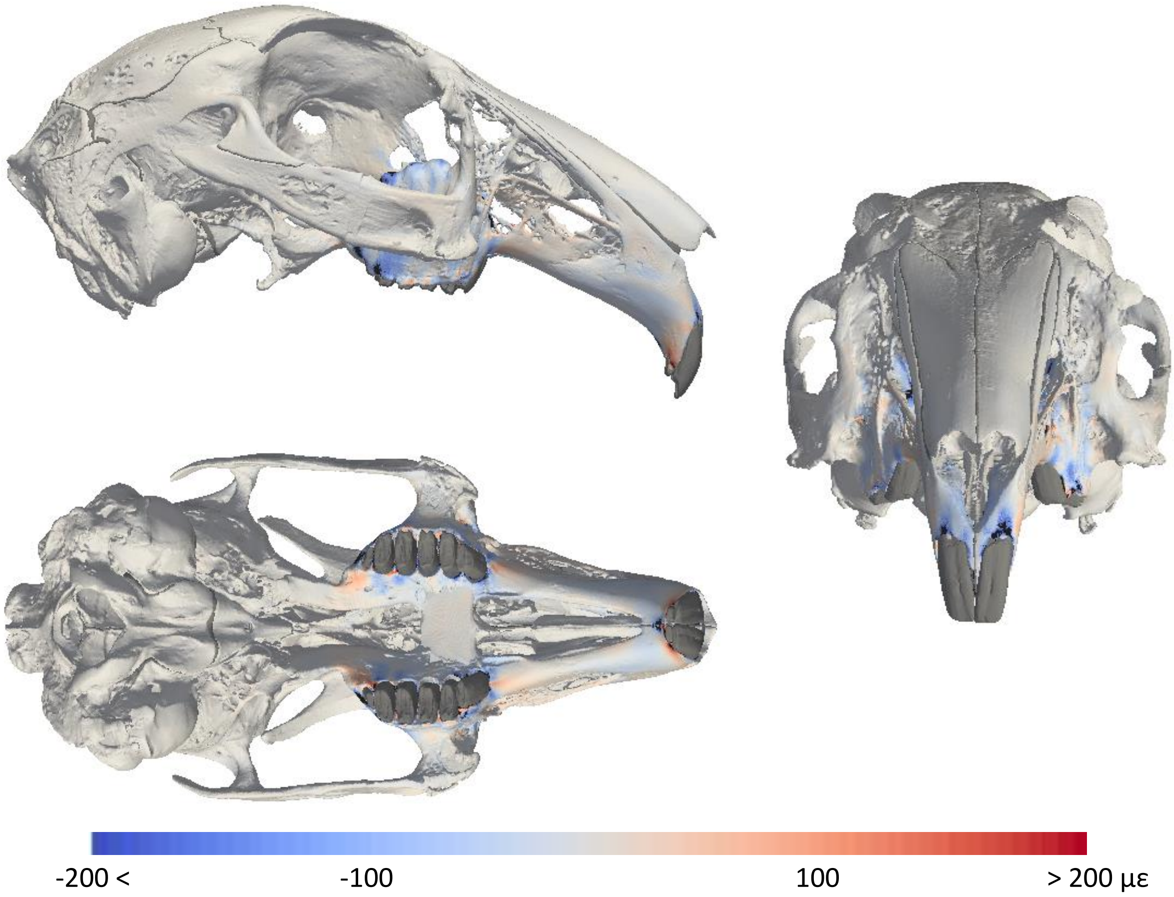

Figure S2. Difference in peak strains across the cranial surface when altering the Young's modulus of the PDL. Positive values are displayed as warm colours, and represent regions where peak strains are larger when modelling E = 50 MPa. Negative values are displayed as cold colours, and represent regions where peak strains are larger when modelling E = 8 MPa. Regions in black represent differences greater than ±200 με, regions in light grey represent minimal difference. Teeth and sutures are shown in dark grey.

To investigate the influence of the suture material properties on bone strain across the cranium, all finite element analyses (one for each of the incisor and molar bites) were re-run with a suture E of 10 MPa (ν = 0.3). The distribution of peak strains in the bone when modelling the sutures with E = 10 MPa (Fig. S3) was comparable with that of the peak strains predicted when modelling E = 20 MPa.

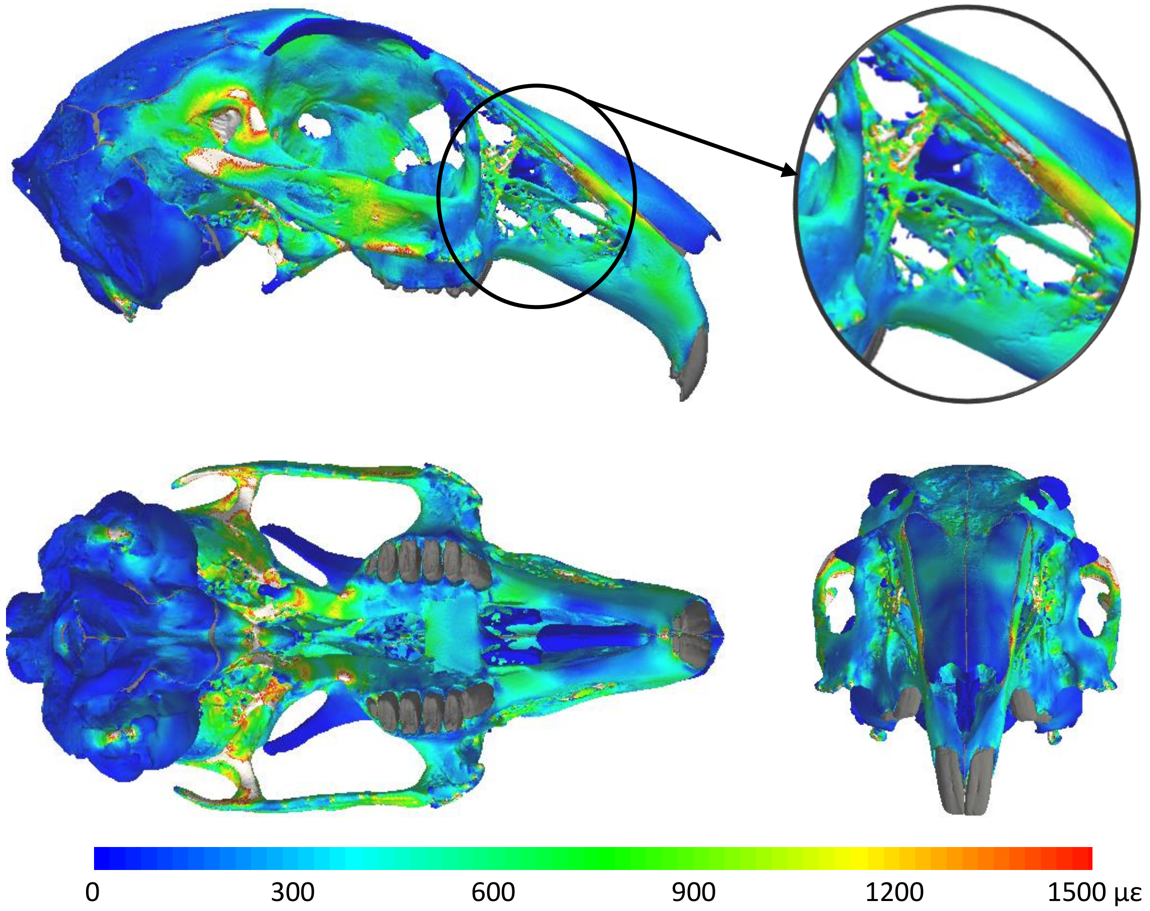

Figure S3. Peak von Mises strain in each element from 12 separate unilateral bites (2 incisor and 10 molar bites) when modelling the sutures with E = 10 MPa (ν = 0.3). The plots show the largest strain in each element from any of the separate bites. The insert shows the rostrum. Regions in grey represent strains above 1500 με, teeth and sutures are shown in dark grey.

Comparison of the difference in peak strains when altering E from 20 MPa to 10 MPa, once again showed a varied distribution in regions which experienced either an increase or decrease in strain magnitude (Fig. S4). These regions generally experience differences below 200 με. However, the remaining regions of the cranium experienced little change in peak strains.

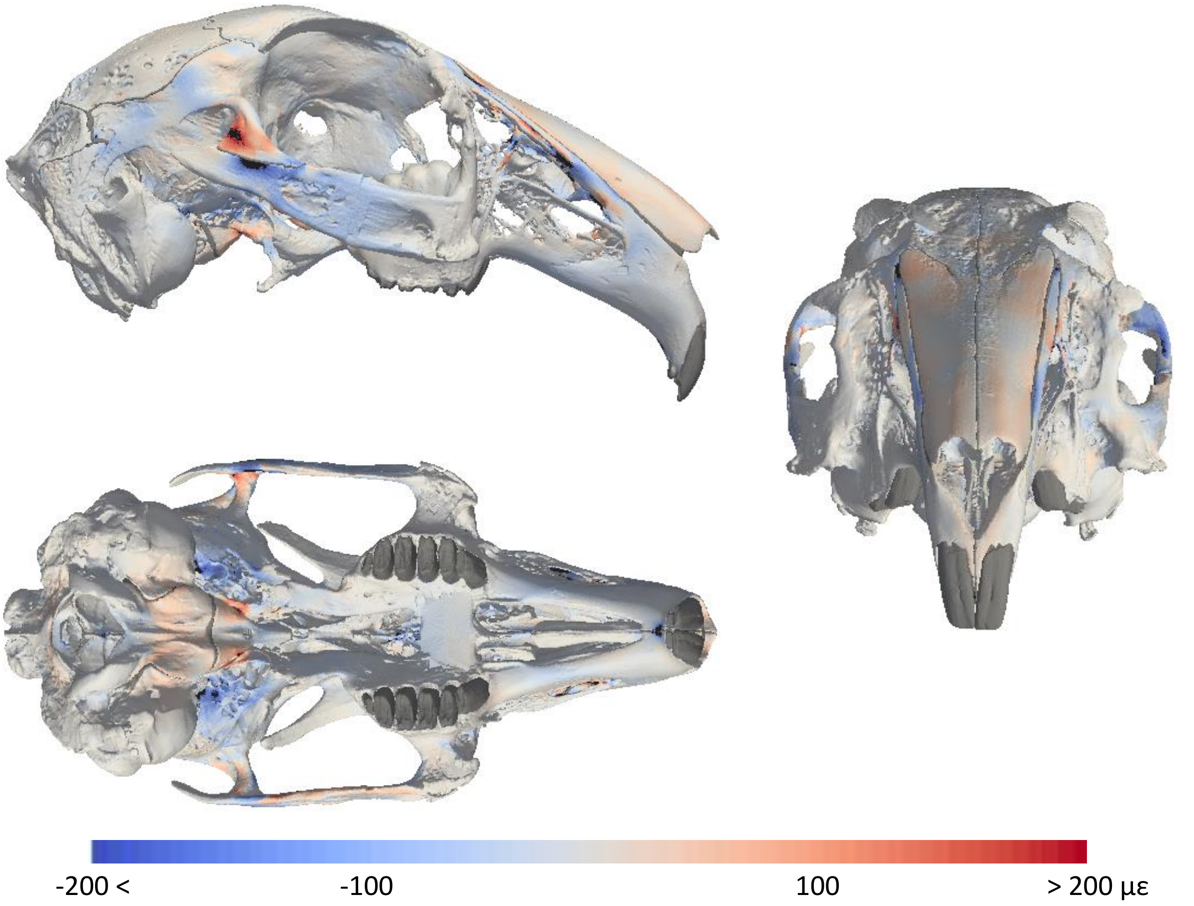

Figure S4. Difference in peak strains across the cranial surface when altering the Young's modulus of the sutures. Positive values are displayed as warm colours, and represent regions where peak strains are larger when modelling E = 20 MPa. Negative values are displayed as cold colours, and represent regions where peak strains are larger when modelling E = 10 MPa. Regions in black represent differences greater than ±200 με, regions in light grey represent minimal difference. Teeth and sutures are shown in dark grey.

A loading regime was created for each bite simulated by the MDA model through using the force calculated in each muscle strand at the point when the bite force reached a maximum value. These muscle forces are displayed in Table S1.

Table S1. The muscle forces calculated by the MDA model for each bite at the point when the bite force reached a maximum value. Muscles were modelled through several strands and the data in this table is the sum of the strand forces for each muscle. The data is taken from bite simulations with the right-hand side as the working side. Muscle forces are displayed in Newtons (N).

	Incisor		1st Premolar		2nd Premolar		1st Molar		2nd Molar		3rd Molar	
	Left	Right	Left	Right	Left	Right	Left	Right	Left	Right	Left	Right
Superficial masseter	7.0	7.4	0.2	11.6	0.1	11.7	0.7	8.7	0.7	9.3	0.4	8.7
Anterior deep masseter	4.4	4.5	0.1	7.1	0.1	7.2	0.4	5.4	0.5	5.7	0.2	5.3
Posterior deep masseter	9.8	8.5	10.3	0.2	10.3	0.1	10.3	0.8	10.3	0.8	10.3	0.4
Anterior zygomaticomandibularis	6.7	4.8	10.5	0.1	10.6	0.1	7.9	0.5	8.4	0.5	7.9	0.3
Posterior zygomaticomandibularis	8.4	6.9	10.5	0.2	10.5	0.2	9.7	0.7	10.0	0.7	9.7	0.4
Superficial temporalis	2.8	2.2	2.8	0.7	2.8	0.7	2.8	0.8	2.8	0.8	2.8	0.8
Medial deep temporalis	2.1	2.1	2.1	0.1	2.1	0.0	2.1	0.2	2.1	0.2	2.1	0.1
Lateral deep temporalis (ventral head)	20.6	21.5	0.6	22.9	0.2	22.9	2.6	22.1	2.6	22.3	1.3	22.1
Lateral deep temporalis (dorsal head)	8.3	8.3	8.3	0.3	8.3	0.1	8.2	1.1	8.2	1.1	8.2	0.6
Medial pterygoid	19.7	21.1	0.5	33.2	0.2	33.5	2.0	25.0	2.0	26.7	1.0	24.8
Lateral pterygoid (superior)	0.7	0.8	0.6	1.2	0.6	1.2	0.7	0.9	0.7	1.0	0.6	0.9
Lateral pterygoid (inferior)	0.6	0.6	1.7	1.0	1.7	1.0	1.8	0.7	1.8	0.8	1.7	0.7
